# Interactions of *CDKAL1* rs7747752 polymorphism and serum levels of L-carnitine and choline are related to increased risk of gestational diabetes mellitus

**DOI:** 10.1186/s12263-022-00716-9

**Published:** 2022-10-01

**Authors:** Hui Wang, Jing Li, Jinnan Liu, Junhong Leng, Weiqin Li, Zhijie Yu, Claudia H. T. Tam, Gang Hu, Ronald C. W. Ma, Zhongze Fang, Ying Wang, Xilin Yang

**Affiliations:** 1 Department of Epidemiology and Biostatistics, School of Public Health, Tianjin Medical University, Tianjin, 300070 China; 2 Tianjin Key Laboratory of Environment, Nutrition and Public Health, Tianjin Medical University, Tianjin, 300070 China; 3 Tianjin Center for International Collaborative Research on Environment, Nutrition and Public Health, Tianjin Medical University, Tianjin, 300070 China; 4 Project Office, Tianjin Women and Children’s Health Center, Tianjin, 300041 China; 5 Population Cancer Research Program and Department of Pediatrics, Dalhousie University, Halifax, B3H 4R2 Canada; 6 Department of Medicine and Therapeutics and Li Ka Shing Institute of Health Sciences, Prince of Wales Hospital, The Chinese University of Hong Kong, Hong Kong, 999077 China; 7 Chronic Disease Epidemiology Laboratory, Pennington Biomedical Research Center, Baton Rouge, Louisiana 70808 USA; 8 Department of Toxicology and Sanitary Chemistry, School of Public Health, Tianjin Medical University, Tianjin, 300070 China; 9 Scientific Research Platform of the Second School of Clinical Medicine & Key Laboratory of 3D Printing Technology in Stomatology, Guangdong Medical University, Dongguan, 523808 Guangdong China

**Keywords:** *CDKAL1*, rs7747752, L-carnitine, Choline, Interaction, Gestational diabetes mellitus

## Abstract

**Background:**

Interactions between genetic, metabolic, and environmental factors lead to gestational diabetes mellitus (GDM). We aimed to examine interactive effects of cyclin-dependent kinase 5 regulatory subunit-associated protein1-like 1(*CDKAL1)* rs7747752 polymorphism with low serum levels of L-carnitine, choline, and betaine for GDM.

**Methods:**

A nested case-control study of 207 GDM women and their one-to-one, age-matched controls was organized from a prospective cohort of pregnant women in Tianjin, China. Conditional logistic regressions were used to test associations between *CDKAL1* rs7747752 and serum levels of L-carnitine, choline, and betaine, and the risk of GDM. Additive interactions were performed to examine interactive effects of rs7747752 and low serum levels of L-carnitine, choline, and betaine on the risk of GDM.

**Results:**

The *CDKAL1* rs7747752 G > C was associated with GDM in additive, dominant, and recessive model (*P* <0.05). The rs7747752 CC genotype enhanced the OR of L-carnitine ≤ vs. > 150 nmol/mL for GDM from 6.14 (2.61–14.4) to 19.6 (5.65–68.1) and the OR of choline ≤ vs. > 110 nmol/mL from 2.37 (1.07–5.28) to 12.1 (3.22–45.6), with significant additive interactions. Similarly, CG genotype also enhanced the OR of L-carnitine ≤ vs. > 150 nmol/mL for GDM from 4.70 (2.01–11.0) to 11.4 (3.98–32.9), with a significant additive interaction. However, the additive interaction between rs7747752 and betaine ≤ 200 nmol/mL on the risk of GDM was not significant.

**Conclusions:**

The CC or CG genotype carriers in rs7747752 of *CDKAL1* who have a low serum level of L-carnitine or choline are at a particular high risk of GDM. Randomized controlled trials are warranted to test the effect of supplement of L-carnitine or choline on the risk of GDM in the high-risk group.

**Supplementary Information:**

The online version contains supplementary material available at 10.1186/s12263-022-00716-9.

## Introduction

With increasing prevalence globally, gestational diabetes mellitus (GDM) has become one of the most common metabolic disorders in pregnancy [1]. GDM leads to adverse short- and long-term consequences for mothers and their offspring, including type 2 diabetes mellitus (T2DM) and cardiovascular diseases in the mothers, as well as macrosomia and childhood obesity in their offspring [2–4]. On the other hand, intensive management of GDM during pregnancy did not have a detectable effect on postpartum diabetes and prediabetes in the mothers [5] and childhood obesity in their offspring [6, 7]. Our meta-analysis showed that lifestyle intervention within 15 weeks of gestation only achieved a 20% reduction in the risk of GDM [8]. Indeed, how to prevent GDM remains one of the priorities in diabetes research.

GDM is a complex disease caused by interactions between genetic, metabolic, and environmental factors [9]. Previous studies have identified some genetic high susceptibilities to GDM [10, 11]. The cyclin-dependent kinase 5 regulatory subunit-associated protein1-like 1 (*CDKAL1*) gene is located at the short arm of human chromosome 6 and encodes a 65 kDa protein. *CDKAL1*, as a mechanism-related protein for diabetes [12], is related to the defects of proinsulin conversion and insulin response under glucose stimulation [13, 14]. Several studies have examined associations between *CDKAL1* and GDM, but their findings are inconsistent. In European populations, *CDKAL1* polymorphisms, i.e., rs7754840 and rs10946398, were not associated with the risk of GDM [15, 16]. On the other hand, *CDKAL1* polymorphisms were strongly associated with risk of GDM in Chinese and other Asian populations [17, 18]. Single nucleotide polymorphism (SNP) rs7747752 is in the intron of *CDKAL1*. Wang et al [10] validated the association between rs7747752 and the risk of GDM in Chinese pregnant women.

Choline, betaine, and L-carnitine, as important metabolic and nutritional factors, are abundant in a wide variety of foods and play a critical role in several physiological processes, such as neurotransmitter synthesis, cell-membrane signaling, and lipid transport [19]. Several studies have attempted to address associations between abnormal levels of these compounds and the risk of diabetes but their findings are inconsistent. Both dietary and serum choline are reported to be positively associated with the risk of T2DM, but betaine is not [20, 21]. Small randomized controlled trials showed that high intake of dietary choline and betaine [22] and oral supplement of carnitine [23] improved insulin resistance in the general population. In this connection, our group observed that low serum levels of L-carnitine, choline, and betaine in early pregnancy were independently associated with markedly increased risk of GDM [24]. The interaction between genetic predisposition and metabolic factors plays a critical role in the development of GDM [25]. Indeed, the *CDKAL1* gene has a strong association with GDM. Importantly, *CDKAL1* genetic variants are predictive of GDM and related glycemic traits [26], suggesting that it may have a synergistic effect with other risk factors. In this regard, our group reported that *CDKAL1* genetic variant had a significant synergistic effect with palmitic acids on the risk of GDM [27]. In addition, animal studies showed that maternal betaine supplementation enhanced lipid metabolism and improved insulin resistance in mice fed a high-fat diet [28, 29]. Additionally, carnitine was able to improve hyperlipidemia, insulin-dependent diabetes mellitus, insulin resistance, and obesity [30]. Given the important role of *CDKAL1* in proinsulin conversion and insulin resistance, it is of interest to explore whether *CKDAL1* genetic variants and low serum levels of L-carnitine, choline, and betaine have synergistic effects on the risk of GDM in Chinese pregnant women.

We organized an age-matched case-control study from a large population-based cohort of pregnant women in Tianjin, China. This analysis aimed to explore additive interactions between rs7747752 and low serum levels of L-carnitine, choline, and betaine for the risk of GDM.

## Materials and methods

### Research design and population

The design, cohort, and methods of this study were described in detail before [31]. In brief, a total of 22,302 pregnant women were recruited into a prospective cohort at their first antenatal care visit through a universal screening and management system for GDM from October 2010 to August 2012. Upon enrollment, they were followed up longitudinally from the first prenatal care till the postpartum period. Ethics of the study protocol was approved by the Ethics Committee of Tianjin Women and Children’s Health Center (TWCHC). Written informed consent was obtained from participants before data collection.

Among the recruited participants, a two-step screening procedure was used to identify GDM. First, a 1-h 50-g glucose challenge test (GCT) in a non-fasting state was performed on pregnant women at 24–28 weeks of gestation at a primary hospital. Women with GCT ≥ 7.8 mmol/L were referred to the GDM clinic in TWCHC for a 2-h 75-g oral glucose tolerance test (OGTT) in the morning after fasting for at least 8 hours. GDM was diagnosed using the International Association of Diabetes and Pregnancy Study Group’s criteria, i.e., a fasting plasma glucose (PG) ≥ 5.1 mmol/L, a 1-h PG ≥ 10.0 mmol/L, or a 2-h PG ≥ 8.5 mmol/L [32].

From July 2011 to June 2012, 2991 pregnant women donated their fasting blood samples overnight at the primary care hospitals. Of them, we excluded 227 women who lacked GCT results or lacked OGTT results when their GCT was ≥ 7.8 mmol/L. Among the remaining 2764 women, a total of 243 GDM women were treated as the cases, and 243 non-GDM women were selected as the controls of the nested case-control study matched by maternal age (±1 year) [33]. After excluding 16 women with low ability of deoxyribonucleic acid (DNA) extraction, the blood samples of 470 women underwent whole-genome sequencing. After further excluding 23 women who lacked high-quality DNA data [34] and 33 women who did not have an age-matched case or control, 414 subjects (207 GDM women and 207 non-GDM women) were included in this study. The flowchart of the study participants was available elsewhere [27].

### Data collection procedures

Data were collected at the first antenatal care visit and at the GCT/OGTT time. The detailed methods of data collection were previously published [31]. Briefly, the data were collected from pregnant women by a series of self-administered questionnaires or retrieved from the database of Maternal and Child Health Information System, including age, ethnicity, education attainment, parity, family history of diabetes in first-degree relatives, and smoking and drinking habits before and during pregnancy. Anthropometric and clinical measurements were performed to collect body weight, height, and systolic/diastolic blood pressure (SBP/DBP). Body mass index (BMI) was estimated as body weight in kilograms divided by the square of body height in meters. The difference in body weight (weight gain) between the first antenatal care visit and GCT was also determined.

### Measurement of serum levels of L-carnitine/choline/betaine

Liquid chromatography-tandem mass spectrometry (LC–MS/MS) was used to assay the serum concentrations of L-carnitine, choline, and betaine. Details of measurement of serum levels of L-carnitine, choline, and betaine were available elsewhere [24].

### Genotyping

DNA samples were genotyped by the Illumina Infinium® Global Screening Array. Genotyping data from specific candidate SNP (rs7747752) were extracted from the genome-wide genotyping. The genotype data were imputed using minimac 3 with the 1000 Genomes Project phase 3 V.5 as a reference panel. The overall genotype call rate was 99.4%.

### Statistical analysis

All statistical analyses were performed using the Statistical Analysis System (SAS) release 9.4 (SAS Institute, Cary, NC). Quantitative data were compared between the GDM group and the non-GDM group with the paired Student’s *t* test or Wilcoxon signed-rank test. The categorical data were compared with McNemar test or Fisher’s exact test. In this analysis, a *P* value < 0.05 was considered to be statistically significant.

Conditional logistic regression was performed to obtain the odds ratios (ORs) and 95% confidence intervals (CIs) of *CDKAL1* rs7747752 genetic variant and serum levels of L-carnitine, choline, and betaine for the risk of GDM. In our previous analyses, we reported that betaine ≤ 200 nmol/mL and choline ≤ 110 and > 270 nmol/mL vs. > 110 to ≤ 270 nmol/mL were independently associated with the increased risk of GDM [24, 35]. In this analysis, we replotted the OR curve of L-carnitine for GDM (Additional file 1: Fig. S1) and refined the selection of the cutoff point of L-carnitine for GDM at ≤ 150 nmol/mL. We tested additive interactions between the rs7747752 genotypes and low serum levels of L-carnitine, choline, and betaine on the risk of GDM. Three measures, i.e., relative excess risk due to the interaction (RERI), attributable proportion due to the interaction (AP), and synergy index (SI), were used to judge the statistical significance of additive interactions [36]. The additive interaction was considered to be significant if any of the following items was statistically significant: RERI > 0, AP > 0, or SI > 1. To control the confounding effects of traditional GDM risk factors, we adjusted for traditional risk factors that included pre-pregnancy BMI, family history of diabetes in first-degree relatives, SBP, current smoker before pregnancy, gestational weeks at GCT, and weight gain to GCT in multivariable model 1, and further adjusted for choline at ≤ 110 vs. > 110 nmol/mL and > 270 vs. ≤ 270 nmol/mL, betaine ≤ 200 nmol/mL, and L-carnitine ≤ 150 nmol/mL (except for testing of its own significance) in multivariable model 2. In addition, we further adjusted for rs7747752 CC genotype (CC vs. CG/GG) in analyses of the additive interaction between rs7747752 genotype (CG vs. GG) and low serum levels of metabolisms for the risk of GDM and further adjusted for rs7747752 CG genotype (CG vs. CC/GG) in analyses of the additive interaction between rs7747752 genotype (CC vs. GG) and low serum levels of metabolisms for the risk of GDM.

## Results

### Characteristics of the participants

The mean age of the participants was 29.2 (±3.0) years, and the mean gestational age was 10.1 (±2.1) weeks at their first antenatal care visit. There were no significant differences between the GDM group and the non-GDM group in terms of height, ethnicity, education attainment, parity, current smoker and alcohol drinker before pregnancy, gestational weeks at GCT, and weight gain from registration to GCT. However, women with GDM had higher values of body weight, BMI, and SBP/DBP at registration than women without GDM. In the GDM group, the proportion of women with a family history of diabetes in first-degree relatives was also higher than that in the non-GDM group. Compared with the non-GDM group, serum levels of choline, L-carnitine, and betaine were lower in the GDM group. The frequencies of the CG and CC genotypes of rs7747752 were found to be significantly higher in women with GDM than in non-GDM women (Table 1).

**Table 1:** Clinical and biochemical characteristics of GDM and non-GDM women

Characteristic	Non-GDM (*n* = 207)	GDM (*n* = 207)	*P*
	mean ± SD/*n* (percentages)	mean ± SD/*n* (percentages)	
**Variables at registration**			
Age, years	29.23±3.34	29.25±2.74	0.480*
Height, cm	162.98±4.54	163.25±5.04	0.509*
Weight, kg	58.58±9.78	63.87±10.54	<0.001*
BMI, kg/m^2^	22.04±3.46	23.95±3.66	<0.001*
Systolic blood pressure, mmHg	104.21±10.60	108.21±10.54	<0.001*
Diastolic blood pressure, mmHg	67.91±7.66	70.72±7.93	<0.001*
Han ethnicity	200 (96.62)	202 (97.58)	0.564**
Education > 12 years	113 (54.59)	109 (52.66)	0.683**
Parity ≥ 1	10 (4.83)	13 (6.28)	0.532**
Family history of diabetes in first-degree relatives	13 (6.28)	26 (12.56)	0.033**
Current smoker before pregnancy	13 (6.28)	14 (6.76)	0.841**
Alcohol drinker before pregnancy	52 (25.12)	63 (30.43)	0.564**
Gestational age, weeks	10.11±2.07	10.12±2.12	0.956*
**Variables during pregnancy**			
Current smoker during pregnancy	1 (0.48)	2 (0.97)	0.564**
Alcohol drinker during pregnancy	2 (0.97)	2 (0.97)	1.000**
Gestational weeks at GCT, weeks	25.16±2.28	24.95±1.44	0.033**
Weight gain to GCT, kg/week	0.58±0.21	0.56±0.23	0.532**
**Metabolites**			
Betaine ≤ 200 nmol/mL	16 (7.73)	64 (30.92)	<0.001**
L-carnitine ≤ 150 nmol/mL	40 (19.32)	143 (69.08)	<0.001**
Choline			<0.001**
≤ 110 nmol/mL	45 (21.74)	100 (48.31)	
> 110 to ≤ 270 nmol/mL	148 (71.50)	89 (43.00)	
> 270 nmol/mL	14 (6.76)	18 (8.70)	
**Genetic variants**			
rs7747752(C/G)			0.003**
GG	64 (30.92)	43 (20.77)	
CG	101 (48.79)	103 (49.76)	
CC	42 (20.29)	61 (29.47)	

*Abbreviations: GDM* gestational diabetes mellitus, *SD* standard deviation, *BMI* body mass index, *GCT* glucose challenge test

*Derived from paired t-test or Wilcoxon signed-rank test

**Derived from the McNemar test or Fisher’s exact test

### Associations of the CDKAL1 rs7747752 and serum levels of L-carnitine, choline, and betaine with the risk of GDM

Serum levels of L-carnitine were negatively associated with the risk of GDM in a non-linear manner (Additional file 1: Fig. S1). L-carnitine ≤ 150 nmol/mL was associated with a markedly increased risk of GDM (OR: 5.73, 95% CI: 2.96–11.1) in multivariable model 2. Similarly, choline ≤ 110 and > 270 nmol/mL vs. > 110 to ≤ 270 nmol/mL (OR: 2.82, 95% CI: 1.44–5.53; OR*:* 4.21, 95% CI: 1.42–12.5) were all associated with elevated risks of GDM in multivariable model 2. Betaine ≤200 nmol/mL was also associated with an increased risk of GDM (OR: 6.02, 95% CI: 2.87–12.6) in multivariable model 1.

In our cohort, the frequency of C allele for rs7747752 genetic variant was 49.89%. The *CDKAL1* rs7747752 C allele was significantly higher in pregnant women with GDM than controls in univariable analysis (OR: 1.48, 95% CI: 1.12–1.96) and after further adjustment for traditional risk factors (OR: 1.74, 95% CI: 1.25–2.42). The CC genotype of rs7747752 was associated with an increased risk of GDM in univariable analysis (OR: 2.19, 95% CI: 1.24–3.85) and after adjustment for traditional risk factors (OR: 2.99, 95% CI: 1.54–5.81). The CG genotype of rs7747752 was also associated with an elevated risk of GDM after adjustment for traditional risk factors (OR: 2.06, 95% CI: 1.20–3.53). Likewise, as compared with GG genotype, CC/CG genotype carriers presented with higher GDM susceptibility in univariable analysis (OR: 1.68, 95% CI: 1.08–2.62) and after adjustment for traditional risk factors (OR: 2.28, 95% CI: 1.35–3.83). As compared with CG/GG genotype, the homozygous CC genotype conferred a 1.63-fold risk of GDM in univariable analysis (OR: 1.63, 95% CI: 1.04–2.57) and a 1.79-fold risk of GDM after adjustment for traditional risk factors (OR: 1.79, 95% CI: 1.06–3.00) (Table 2).

**Table 2:** Odds ratios of low L-carnitine, choline, and betaine and rs7747752 for gestational diabetes mellitus

	Multivariable model 1		Multivariable model 2	
	OR (95% CI)	*P*	OR (95% CI)	*P*
**Metabolites**				
Betaine ≤ vs. > 200 nmol/mL	6.02 (2.87–12.6)	<0.001	2.31 (0.93–5.76)	0.073
L-carnitine ≤ vs. > 150 nmol/mL	8.27 (4.53–15.1)	<0.001	5.73 (2.96–11.1)	<0.001
Choline				
≤ 110 nmol/mL	4.63 (2.60–8.24)	<0.001	2.82 (1.44–5.53)	0.003
> 110 to ≤ 270 nmol/mL	1.00	-	1.00	-
> 270 nmol/mL	2.86 (1.18–6.94)	0.020	4.21 (1.42–12.5)	0.009
**Genetic variant**^a^				
rs7747752				
GG	1.00	-	1.00	-
CG	1.51 (0.95–2.41)	0.080	2.06 (1.20–3.53)	0.009
CC	2.19 (1.24–3.85)	0.007	2.99 (1.54–5.81)	0.001
Additive (CC vs. CG vs. GG)	1.48 (1.12–1.96)	0.007	1.74 (1.25–2.42)	0.001
Dominant (CC/CG vs. GG)	1.68 (1.08–2.62)	0.023	2.28 (1.35–3.83)	0.002
Recessive (CC vs. CG/GG)	1.63 (1.04–2.57)	0.034	1.79 (1.06–3.00)	0.029

*Abbreviations:* *OR* odds ratio, *CI* confidence interval

Multivariable model 1, adjusted for traditional risk factors, including pre-pregnancy BMI, family history of diabetes in first-degree relatives, systolic blood pressure, current smoker before pregnancy, gestational weeks at glucose challenge test, and weight gain to the time of glucose challenge test

Multivariable model 2, adjusted for choline at ≤ 110 vs. > 110 nmol/mL and ≤ 270 vs. > 270 nmol/mL, betaine ≤ 200 nmol/mL, and L-carnitine ≤ 150 nmol/mL (except for testing of its own significance), in addition to the variables listed in multivariable model 1

^a^Only tested its own significance and included no other variables in the multivariable model 1 and adjusted for traditional risk factors that included pre-pregnancy BMI, family history of diabetes in first-degree relatives, systolic blood pressure, current smoker before pregnancy, gestational weeks at glucose challenge test, and weight gain to the time of glucose challenge test in multivariable model 2

### Associations between combinations of rs7747752 genotypes (CC vs. CG vs. GG) and low serum levels of L-carnitine, choline, and betaine for the risk of GDM

Among the combinations of *CDKAL1* rs7747752 genotypes and low serum levels of L-carnitine, L-carnitine ≤ 150 nmol/mL combined with CG genotype was associated with an increased risk of GDM after adjustment for traditional GDM risk factors as well as low & high choline and low betaine (OR: 9.90, 95% CI: 3.12–31.4). L-carnitine ≤ 150 nmol/mL combined with CC genotype was also associated with an increased risk of GDM (OR: 14.6, 95% CI: 3.84–55.9). Among the combinations of rs7747752 genotypes and choline ≤ 110 nmol/mL, choline ≤ 110 nmol/mL and CG genotype was associated with an elevated risk of GDM after adjustment for traditional GDM risk factors as well as low L-carnitine and low betaine (OR: 4.30, 95% CI: 1.49–12.4). Choline ≤ 110 nmol/mL and CC genotype were associated with an increased risk of GDM (OR: 12.4, 95% CI: 3.10–49.2). Similarly, the combination of betaine ≤ 200 nmol/mL and CC genotype was also associated with an increased risk of GDM after adjustment for traditional GDM risk factors as well as low & high choline and low L-carnitine (OR: 9.18, 95% CI: 1.54–54.7) (Table 3).

**Table 3:** Combination effects of rs7747752 and low L-carnitine, choline, and betaine for gestational diabetes mellitus

SNP	Metabolites	Non-GDM (*n* = 207)	GDM (*n* = 207)	Multivariable model 1		Multivariable model 2	
				OR (95% CI)	*P*	OR (95% CI)	*P*
**Combinations of rs7747752 genotypes and L-carnitine**							
rs7747752	L-carnitine (in nmol/mL)						
GG	>150	51 (24.64)	12 (5.80)	1.00	-	1.00	-
GG	≤150	13 (6.28)	31 (14.98)	6.09 (2.10–17.6)	<0.001	3.57 (1.00–12.7)	0.050
CG	>150	81 (39.13)	29 (14.01)	1.22 (0.48–3.08)	0.676	1.18 (0.43–3.25)	0.744
CG	≤150	20 (9.66)	74 (35.75)	15.9 (5.91–42.9)	<0.001	9.90 (3.12–31.4)	<0.001
CC	>150	35 (16.91)	23 (11.11)	2.69 (0.96–7.53)	0.060	2.45 (0.78–7.70)	0.125
CC	≤150	7 (3.38)	38 (18.35)	17.2 (5.02–58.9)	<0.001	14.6 (3.84–55.9)	<0.001
**Combinations of rs7747752 genotypes and choline**							
rs7747752	Choline (in nmol/mL)						
GG	>110	47 (22.71)	21 (10.14)	1.00	-	1.00	-
GG	≤110	17 (8.21)	22 (10.63)	5.11 (1.79–14.6)	0.002	2.51 (0.68–9.27)	0.167
CG	>110	82 (39.61)	53 (25.61)	2.03 (0.98–4.22)	0.057	1.85 (0.76–4.54)	0.178
CG	≤110	19 (9.18)	50 (24.15)	9.04 (3.70–22.1)	<0.001	4.30 (1.49–12.4)	0.007
CC	>110	33 (15.94)	33 (15.94)	3.31 (1.38–7.96)	0.008	2.84 (1.02–7.90)	0.045
CC	≤110	9 (4.35)	28 (13.53)	16.6 (4.97–55.5)	<0.001	12.4 (3.10–49.2)	<0.001
**Combinations of rs7747752 genotypes and betaine**							
rs7747752	Betaine (in nmol/mL)						
GG	>200	59 (28.50)	26 (12.56)	1.00	-	1.00	-
GG	≤200	5 (2.42)	17 (8.21)	9.16 (2.38–35.2)	0.001	3.45 (0.65–18.3)	0.145
CG	>200	93 (44.93)	73 (35.27)	2.26 (1.18–4.31)	0.013	1.97 (0.90–4.34)	0.092
CG	≤200	8 (3.86)	30 (14.49)	8.65 (3.07–24.4)	<0.001	3.76 (0.98–14.4)	0.053
CC	>200	39 (18.84)	44 (21.26)	3.28 (1.43–7.52)	0.005	3.69 (1.36–10.0)	0.010
CC	≤200	3 (1.45)	17 (8.21)	28.8 (6.17–134)	<0.001	9.18 (1.54–54.7)	0.015

*Abbreviations:* *SNP* single nucleotide polymorphism, *GDM* gestational diabetes mellitus, *OR* odds ratio, *CI* confidence interval

Multivariable model 1, adjusted for traditional risk factors, including pre-pregnancy BMI, family history of diabetes in first-degree relatives, systolic blood pressure, current smoker before pregnancy, gestational weeks at glucose challenge test, and weight gain to the time of glucose challenge test

Multivariable model 2, adjusted for choline at ≤ 110 vs. > 110 nmol/mL and ≤ 270 vs. > 270 nmol/mL, betaine ≤ 200 nmol/mL, and L-carnitine ≤ 150 nmol/mL (except for testing of its own significance), in addition to the variables listed in multivariable model 1

### Additive interactions between rs7747752 genotypes (CC/CG vs. GG) and low serum levels of L-carnitine, choline, and betaine for the risk of GDM

The CG genotype of rs7747752 greatly enhanced the OR (95% CI) of L-carnitine ≤ vs. > 150 nmol/mL for GDM from 6.23 (2.89–13.5) to 16.1 (6.26–41.3) in multivariable model 1 and from 4.70 (2.01–11.0) to 11.4 (3.98–32.9) in multivariable model 2. The AP was 0.60 (0.25–0.94) and 0.56 (0.15–0.97), respectively (Table 4). However, the additive interactions between rs7747752 CG and low choline and between rs7747752 CG and low betaine for the risk of GDM were nonsignificant (Tables 4 and 5).

**Table 4:** Additive interactions between rs7747752 and low L-carnitine and choline for gestational diabetes mellitus

SNP	Metabolites	Multivariable model 1		Multivariable model 2	
		OR/estimate	*P*	OR/estimate	*P*
		(95% CI)		(95% CI)	
**Additive interaction between rs7747752 genotypes CG (vs. GG) and low L-carnitine and choline**^a^					
rs7747752	L-carnitine (in nmol/mL)				
GG	>150	1.00	-	1.00	-
GG	≤150	6.23 (2.89–13.5)	<0.001	4.70 (2.01–11.0)	<0.001
CG	>150	1.23 (0.52–2.92)	0.636	1.34 (0.53–3.38)	0.541
CG	≤150	16.1 (6.26–41.3)	<0.001	11.4 (3.98–32.9)	<0.001
RERI		9.62 (−3.49 to 22.7)		6.40 (−3.82 to 16.6)	
AP		0.60 (0.25–0.94)		0.56 (0.15–0.97)	
SI		2.76 (1.07–7.16)		2.59 (0.88–7.62)	
rs7747752	Choline (in nmol/mL)				
GG	>110	1.00	-	1.00	-
GG	≤110	5.07 (2.33–11.1)	<0.001	3.28 (1.27–8.49)	0.014
CG	>110	2.03 (1.01–4.07)	0.047	2.02 (0.86–4.73)	0.105
CG	≤110	9.01 (3.87–21.0)	<0.001	4.82 (1.79–13.0)	0.002
RERI		2.91 (−3.48 to 9.31)		0.52 (−3.70 to 4.73)	
AP		0.32 (−0.20 to 0.84)		0.11 (−0.71 to 0.92)	
SI		1.57 (0.65–3.82)		1.16 (0.36–3.73)	
**Additive interaction between rs7747752 genotypes CC (vs. GG) and low L-carnitine and choline**^b^					
rs7747752	L-carnitine (in nmol/mL)				
GG	>150	1.00	-	1.00	-
GG	≤150	9.57 (4.65–19.7)	<0.001	6.14 (2.61–14.4)	0.034
CC	>150	3.55 (1.41–8.94)	0.005	3.43 (1.26–9.35)	0.034
CC	≤150	21.7 (6.78–69.3)	<0.001	19.6 (5.65–68.1)	<0.001
RERI		9.55 (−13.0 to 32.1)		11.1 (−10.9 to 33.0)	
AP		0.44 (−0.14 to 1.03)		0.56 (0.06–1.06)	
SI		1.86 (0.62–5.60)		2.46 (0.73–8.35)	
rs7747752	Choline (in nmol/mL)				
GG	>110	1.00	-	1.00	-
GG	≤110	4.65 (2.37–9.12)	<0.001	2.37 (1.07–5.28)	0.047
CC	>110	3.18 (1.42–7.11)	0.005	2.79 (1.08–7.18)	0.037
CC	≤110	16.0 (5.03–50.8)	<0.001	12.1 (3.22–45.6)	0.001
RERI		9.14 (−7.94 to 26.2)		7.94 (−7.28 to 23.2)	
AP		0.57 (0.11–1.03)		0.66 (0.20–1.11)	
SI		2.57 (0.81–8.17)		3.51 (0.77–16.0)	

Multivariable model 2, adjusted for choline at ≤ 110 vs. > 110 nmol/mL and ≤ 270 vs. > 270 nmol/mL, betaine ≤ 200 nmol/mL, and L-carnitine ≤ 150 nmol/mL (except for testing of its own additive interaction), in addition to the variables listed in multivariable model 1

RERI > 0, AP > 0, or SI > 1 indicates a significant additive interaction

*Abbreviations:* *SNP* single nucleotide polymorphism, *OR* odds ratio, *CI* confidence interval, *RERI* relative excess risk due to interaction, *AP* attributable proportion due to interaction, *SI* synergy index

^a^Multivariable model 1, adjusted for traditional risk factors, including pre-pregnancy BMI, family history of diabetes in first-degree relatives, systolic blood pressure, current smoker before pregnancy, gestational weeks at glucose challenge test, and weight gain to the time of glucose challenge test, and further adjusted rs7747752 genotype (CC vs. CG/GG)

^b^Multivariable model 1, adjusted for traditional risk factors, including pre-pregnancy BMI, family history of diabetes in first-degree relatives, systolic blood pressure, current smoker before pregnancy, gestational weeks at glucose challenge test, and weight gain to the time of glucose challenge test, and further adjusted rs7747752 genotype (CG vs. CC/GG)

**Table 5:** Additive interactions between rs7747752 and low betaine for gestational diabetes mellitus

SNP	Metabolites	Multivariable model 1		Multivariable model 2	
		OR/estimate	*P*	OR/estimate	*P*
		(95% CI)		(95% CI)	
**Additive interaction between rs7747752 genotypes CG (vs. GG) and low betaine**^a^					
rs7747752	Betaine (in nmol/mL)				
GG	>200	1.00	-	1.00	-
GG	≤200	9.00 (3.10–26.1)	<0.001	2.99 (0.84–10.7)	0.092
CG	>200	2.25 (1.20–4.21)	0.011	1.91 (0.90–4.06)	0.093
CG	≤200	8.63 (3.08–24.2)	<0.001	3.64 (0.98–13.5)	0.054
RERI		−1.62 (−13.2 to 9.95)		−0.26 (−5.53 to 5.01)	
AP		−0.19 (−1.64 to 1.27)		−0.07 (−1.58 to 1.44)	
SI		0.83 (0.21–3.26)		0.91 (0.13–6.24)	
**Additive interaction between rs7747752 genotypes CC (vs. GG) and low betaine**^b^					
rs7747752	Betaine (in nmol/mL)				
GG	>200	1.00	-	1.00	-
GG	≤200	5.30 (2.37–11.9)	<0.001	2.36 (0.82–6.76)	0.109
CC	>200	2.85 (1.31–6.19)	0.008	3.30 (1.31–8.32)	0.011
CC	≤200	24.6 (5.45–111)	<0.001	8.21 (1.44–46.8)	0.018
RERI		17.4 (−18.4 to 53.3)		3.54 (−10.4 to17.4)	
AP		0.71 (0.28–1.14)		0.43 (−0.57 to 1.44)	
SI		3.83 (0.81–18.1)		1.97 (0.25–15.3)	

Multivariable model 2, adjusted for choline at ≤110 vs. >110 nmol/mL and ≤270 vs. >270 nmol/mL and L-carnitine ≤150 nmol/mL, in addition to the variables listed in multivariable model 1

RERI >0, AP >0, or SI >1 indicates a significant additive interaction

*Abbreviations:* *SNP* single nucleotide polymorphism, *OR* odds ratio, *CI* confidence interval, *RERI* relative excess risk due to interaction, *AP* attributable proportion due to interaction, *SI* synergy index

^a^Multivariable model 1, adjusted for traditional risk factors, including pre-pregnancy BMI, family history of diabetes in first-degree relatives, systolic blood pressure, current smoker before pregnancy, gestational weeks at glucose challenge test, and weight gain to the time of glucose challenge test, and further adjusted rs7747752 genotype (CC vs. CG/GG)

^b^Multivariable model 1, adjusted for traditional risk factors, including pre-pregnancy BMI, family history of diabetes in first-degree relatives, systolic blood pressure, current smoker before pregnancy, gestational weeks at glucose challenge test, and weight gain to the time of glucose challenge test, and further adjusted rs7747752 genotype (CG vs. CC/GG)

The CC genotype of rs7747752 markedly enhanced the OR (95% CI) of L-carnitine ≤ vs. > 150 nmol/mL for GDM from 6.14 (2.61–14.4) to 19.6 (5.65–68.1) and also enhanced the OR of choline ≤ vs. > 110 nmol/mL for GDM from 2.37 (1.07–5.28) to 12.1 (3.22–45.6) in multivariable model 2. Their APs were, respectively, 0.56 (0.06–1.06) and 0.66 (0.20–1.11), being statistically significant (Table 4). However, we failed to detect a significant additive interaction between rs7747752 CC and low serum levels of betaine for the risk of GDM (Table 5).

## Discussion

We verified that *CDKAL1* rs7747752 genetic variant was associated with an elevated risk of GDM in Chinese pregnant women. Interestingly, we, for the first time, found that the *CDKAL1* rs7747752 CC/CG genotype markedly amplified the GDM-promoting effects of low serum levels of L-carnitine and choline, leading to a large increase in the risk of GDM in Chinese women. Unfortunately, we did not find that *CDKAL1* rs7747752 had a significant synergistic effect with betaine on the risk of GDM.

There is a lack of studies that addressed associations of serum L-carnitine, choline, and betaine with the risk of GDM. However, several studies have attempted to address associations between L-carnitine, choline, and betaine and diabetes, but their findings are inconsistent and inconclusive. A cross-sectional study of 7074 Norwegian men and women observed that choline was positively while betaine was negatively associated with key components of metabolic syndrome [37]. A case-control study of 427 pairs of individuals with and without incident diabetes nested in the Diabetes Prevention Program (DPP) found that low plasma betaine at baseline was associated with an increased risk of incident diabetes. It is also noted that the increase in betaine levels at 2 years of follow-up was associated with a lower risk of incident diabetes while higher dietary intakes of L-carnitine, choline, and betaine were associated with a decreased risk of T2DM [38]. A cross-sectional study of 2394 adults in Canada showed that dietary choline and betaine intakes were inversely correlated with the levels of fasting glucose and insulin resistance [22]. However, the Atherosclerosis Risk in Communities (ARIC) study (*n* = 13,440) found that dietary intake of choline was associated with the risk of T2DM in women, but betaine was not [20]. Stronger evidence came from a meta-analysis of 5 randomized controlled trials showing that L-carnitine treatment was effective in reducing insulin resistance over a 12-month period [39]. In our case-control study, low serum levels of L-carnitine, choline, and betaine in early pregnancy were also associated with markedly elevated risks of GDM [24]. In this analysis, we further found that low serum levels of L-carnitine and choline had synergistic effects with the *CDKAL1* rs7747752 CC/CG genotype towards the increasing risk of GDM. This observation provides a plausible explanation for some inconsistent findings regarding associations of L-carnitine and choline with the risk of T2DM, i.e., the effects of the supplement of L-carnitine and choline being particularly large among *CDKAL1* rs7747752 CC/CG genotype carriers who also had a low serum level of L-carnitine or choline.

To our best knowledge, we are the first to report the additive interactions between *CDKAL1* genetic variants and low serum levels of L-carnitine and choline for GDM. *CDKAL1* is identified so far as being significantly associated with T2DM [40]. The *CDKAL1* genetic variants could predict the development of diabetes in individuals with impaired insulin secretion, which indicates that there may be potential synergistic and interactive effects among different risk factors [13, 41]. In a recent study, our group found that *CDKAL1* genetic variant had a significant synergistic effect with serum palmitate acids, leading to an increased risk of GDM [27]. The interaction between the two risk factors may suggest that impaired beta cells cannot produce enough insulin to cope with increased insulin resistance as caused by high palmitate acids, thereby triggering a high risk of GDM [27]. Intriguingly, we further detected significant additive interactions between *CDKAL1* rs7747752 CC/CG genotype and low serum levels of L-carnitine and choline for the risk of GDM. Women who had both *CDKAL1* rs7747752 CC/CG genotype and low serum levels of L-carnitine and choline were at a particularly high risk of GDM.

The molecular mechanism of additive interactions between low serum levels of L-carnitine and choline and *CDKAL1* rs7747752 for the risk of GDM remains unclear. However, it is biologically plausible that L-carnitine and choline play a critical role in the link between *CDKAL1* genetic variants and GDM. Studies suggest that *CDKAL1* polymorphisms may modulate insulin secretion [42] or be related to insulin resistance [43, 44]. Notably, the Cardiovascular Health Study found that high plasma concentrations of choline were associated with decreased fasting glucose and insulin levels and increased insulin sensitivity [45]. Furthermore, Zhang et al. observed that a decrease in serum choline was associated with an increase in insulin resistance [46]. Thus, it is likely that the copresence of low serum levels of choline and *CDKAL1* rs7747752 genetic variant led to a markedly increased risk of GDM via increasing insulin resistance or decreasing insulin sensitivity. A primary physiological role of L-carnitine is to transfer long-chain fatty acids into the mitochondrial matrix and to increase the efflux of acyl groups out of the mitochondria [47]. Accumulation of intracellular lipid derivatives within mitochondria plays an important role in the development of insulin resistance [48]. Several lines of evidence suggest a role of L-carnitine in regulating insulin resistance and glucose metabolism [49–51]. Hence, their interactions for GDM may suggest that GDM develops when impaired beta cells could not produce enough insulin in response to increased insulin resistance as manifested by low L-carnitine. It is also possible that the copresence of both risk factors further contributed to increased insulin resistance, as compared with any one risk factor alone, thereby triggering a high GDM risk status. Hereby, our findings seem to support that low serum levels of L-carnitine and choline among CC or CG genotype carriers at *CDKAL1* rs7747752 play a critical role in the etiology of GDM.

Our findings have potential implications for prevention of GDM. GDM is prevalent in the world, and an increasing number of women are being affected by the disease. What is worse, women with GDM are at a much higher risk of diabetes in later life and their offspring are also at a high risk of obesity in childhood. It is critical to reduce the burden of GDM and its short- and long-term complications. In this connection, our study suggests that pregnant women should be screened for copresence of CC/CG genotypes of rs7747752 in *CDKAL1* and low serum levels of L-carnitine or choline. Specific interventions, such as increased dietary intakes or supplements of L-carnitine or choline, may be used to prevent GDM in CC/CG genotype carriers who had low serum L-carnitine or choline in early pregnancy. Indeed, randomized controlled trials are warranted to test the efficacy of supplements of L-carnitine or choline among pregnant women who have both risk factors in early pregnancy.

This study had several limitations. First, our findings were obtained from a nested case-control study of Chinese pregnant women. Further cohort studies are needed to replicate these important findings in other populations of pregnant women. Second, we used a two-step GDM screening procedure to identify GDM and some GDM cases might have been missed. Third, diagnostic methods of GDM differ from country to country and even from medical organization to medication organization in a single country. These differences might also affect the implacability of our study findings. Fourth, dietary intakes of L-carnitine, choline, and betaine may affect its serum level [52]. However, information on dietary intakes was not collected due to the busy clinical setting. Interactions between high intakes of L-carnitine and choline and *CDKAL1* genetic variants for the risk of GDM need to be further confirmed.

## Conclusion

We found that the *CDKAL1* rs7747752 genetic variant interacted with serum L-carnitine ≤ 150 nmol/mL and choline ≤ 110 nmol/mL, leading to a markedly increased risk of GDM. Identification of interactions of rs7747752 CC/CG genotype with L-carnitine and choline metabolites is an important step towards effectively preventing GDM. Replication studies are needed to confirm our findings in other populations of pregnant women, and further mechanistic studies are also warranted to understand the molecular mechanism of the interactions between *CDKAL1* rs7747752 genetic variant and low levels of L-carnitine and choline for the risk of GDM.

## Supplementary Information

**Additional file 1: Figure S1**. Associations between L-carnitine and the risk of gestational diabetes mellitus (GDM) in Chinese women.

## Data Availability

The datasets used and/or analyzed during the current study are available from the corresponding author on reasonable request.
