# Supplementary material for: Interactions of CDKAL1 rs7747752 polymorphism and serum levels of L-carnitine and choline are related to increased risk of gestational diabetes mellitus
Source: Genes Nutr. 2022 Oct 1;17:14. doi: 10.1186/s12263-022-00716-9 (PMC9526259; doi:10.1186/s12263-022-00716-9)
Supplement: Supplementary file 1 — Additional file 1: Figure S1. Associations between L-carnitine and the risk of gestational diabetes mellitus (GDM) in Chinese women. [file 12263_2022_716_MOESM1_ESM.docx]

**Interactions of *CDKAL1* rs7747752 polymorphism and serum levels of L-carnitine and choline are related to increased risk of gestational diabetes mellitus**

Running title: *CDKAL1* rs7747752, L-carnitine & choline for GDM

Hui Wang (MD) ^a, †^, Jing Li (PhD) ^a, h, i, †^, Jinnan Liu (BA) ^a^, Junhong Leng (PhD) ^b^, Weiqin Li (MD) ^b^, Zhijie Yu (PhD) ^c^, Claudia HT Tam (PhD) ^d^, Gang Hu (PhD) ^e^, Ronald CW Ma (MD, FRCP) ^d^, Zhongze Fang (PhD) ^f, h, i^, Ying Wang (PhD) ^g, *^, Xilin Yang (PhD) ^a, h, i, *^

^†^Equal contribution to the manuscript.


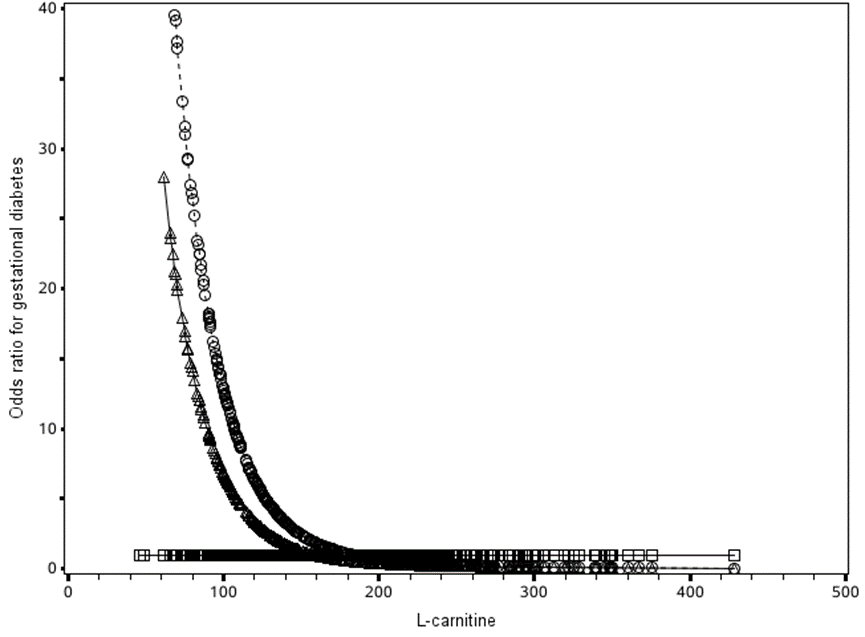


Additional Figure 1.

Title: Associations between L-carnitine and the risk of gestational diabetes mellitus (GDM) in Chinese women.

Legends: The line connecting square is the reference lines at OR=1. The line connecting triangle and the line connecting circular stand for the unadjusted and adjusted ORs for GDM, respectively.
